# Frailty index as a clinical measure of biological age in psychiatry

**DOI:** 10.1192/j.eurpsy.2021.197

**Published:** 2021-08-13

**Authors:** F.S. Bersani

**Affiliations:** Department Of Human Neurosciences, Sapienza University of Rome, Rome, Italy

**Keywords:** comorbidity, accelerated biological aging, frailty index, deficit accumulation model

## Abstract

The concepts of “accelerated biological ageing” and “premature biological senescence” have been receiving increasing attention in relation to psychiatric diseases, with clinical, epidemiological and molecular observations suggesting that psychopathological processes can have significant relationships with aging-related phenomena. The deficit accumulation model postulates that the individual’s biological age and functional status is related to the amount of health deficits accumulated over time and that one’s biological age can be estimated by summarizing health deficits in a single continuous variable, the so-called “frailty index” (FI). In this presentation it will be discussed the possibility that the FI, which condenses information arising from multidimensional evaluations, represents a potential clinically-useful macroscopic indicator of biological age which can add relevant information to the measurements currently implemented in the study of accelerated biological age in psychiatric diseases.

**Disclosure:**

No significant relationships.

